# Chicken cathelicidin-2 promotes NLRP3 inflammasome activation in macrophages

**DOI:** 10.1186/s13567-022-01083-4

**Published:** 2022-09-05

**Authors:** Lianci Peng, Hongliang Tian, Yi Lu, Kaixiang Jia, Jinrong Ran, Qi Tao, Gang Li, Chao Wan, Chao Ye, Edwin J. A. Veldhuizen, Hongwei Chen, Rendong Fang

**Affiliations:** 1 Joint International Research Laboratory of Animal Health and Animal Food Safety, College of Veterinary Medicine, Southwest University, Chongqing, 400715 China; 2 Immunology Research Center, Institute of Medical Research, Southwest University, Chongqing, 402460 China; 3 Department of Biomolecular Health Sciences, Division Infectious Diseases & Immunology, Section Immunology, Faculty of Veterinary Medicine, Utrecht University, Utrecht, The Netherlands

## Abstract

Chicken cathelicidin-2 (CATH-2) as a host defense peptide has been identified to have potent antimicrobial and immunomodulatory activities. Here, we reported the mechanism by which CATH-2 modulates NLRP3 inflammasome activation. Our results show that CATH-2 and ATP as a positive control induced secretion of IL-1β and IL-1α in LPS-primed macrophages but did not affect secretion of IL-6, IL-12 and TNF-α. Furthermore, CATH-2 induced caspase-1 activation and oligomerization of apoptosis-associated speck-like protein containing a carboxy- terminal caspase recruitment domain (ASC), which is essential for NLRP3 inflammasome activation. However, CATH-2 failed to induce IL-1β secretion in Nlrp3^−/−^, Asc^−/−^ and Casp1^−/−^ macrophages. Notably, IL-1β and NLRP3 mRNA expression were not affected by CATH-2. In addition, CATH-2-induced NLRP3 inflammasome activation was mediated by K^+^ efflux but independent of the P2X7 receptor that is required for ATP-mediated K^+^ efflux. Gene interference of NEK7 kinase which has been identified to directly interact with NLRP3, significantly reduced IL-1β secretion and caspase-1 activation induced by CATH-2. Furthermore, confocal microscopy shows that CATH-2 significantly induced lysosomal leakage with the diffusion of dextran fluorescent signal. Cathepsin B inhibitors completely abrogated IL-1β secretion and caspase-1 activation as well as attenuating the formation of ASC specks induced by CATH-2. These results all indicate that CATH-2-induced activation of NLRP3 inflammasome is mediated by K^+^ efflux, and involves the NEK7 protein and cathepsin B. In conclusion, our study shows that CATH-2 acts as a second signal to activate NLRP3 inflammasome. Our study provides new insight into CATH-2 modulating immune response.

## Introduction

Antimicrobial resistance is one of the major challenges for human and animal health in the twenty-first century and new alternative strategies are needed to combat this global problem [1, 2]. So far, discovering new antibiotics based on conventional targets of microorganisms has failed to produce new classes of antibiotics [3, 4]. Therefore, there is an urgent need to find an innovative method to develop new drugs against microbial infections [5, 6]. Boosting host defense to kill pathogens rather than kill pathogens itself could be such a new potential therapy.

Host defense peptides (HDP) also known as antimicrobial peptides, are identified to control infections by directly killing pathogens or by modulating the host immune response [7]. Cathelicidins as one of the main families of HDP, are broadly expressed in different animals and could be developed as potential immunomodulatory molecules to treat microbial infection [8]. Immunomodulatory functions of chicken cathelicidin-2 (CATH-2) has been extensively studied in different species, such as in human, murine and porcine cells [9–15]. It efficiently neutralizes LPS and inhibits Toll like receptor-4 (TLR-4) activation in a non-species specific manner [14, 15]. Furthermore, the treatment of D-analog of CATH-2 in ovo reduced mortality and morbidity in chickens from *E. coli* infection up to 7 days after hatching [16]. These studies indicate that CATH-2 protects the host beyond antimicrobials itself but also modulates immune response and exerts its function cross-species.

Inflammasomes are an intracellular complex of proteins composed of a central protein, an adaptor (ASC) and an effector (caspase-1), which play an important role in the host defense against microbial infection. NOD-, LRR- and pyrin domain-containing protein 3 (NLRP3) are intracellular sensors detecting a broad range of microbial motifs, resulting in assembly and activation of an inflammasome, which finally leads to caspase-1‑mediated IL-1β maturation and secretion [17]. The NLRP3 inflammasome has been demonstrated to be important in response to infection by different pathogens, such as influenza virus, *Streptococcal pneumoniae*, *Candida albicans*, *Pasteurella multocida* and *Staphylococcus aureus* [18–21]. NLRP3 full activation requires two signals for priming and inflammasome formation. In addition, NLRP3 activation is thought to be involved in multiple upstream signals including efflux of potassium ions (K^+^), NIMA-related kinase 7 (NEK7) and lysosomal disruption [17]. It has been reported that human cathelicidin LL-37 can promote NLRP3 activation via the P2X7 receptor [22], but that is the only study that shows involvement of a cathelicidin in inflammasome formation; therefore data on whether other cathelicidins, including chicken CATH-2 can also regulate NLRP3 activation and especially whether the same mechanism is used, is still absent. A previous study has shown that mouse macrophages can be used as a good model to investigate the mechanism by which CATH-2 modulates innate immune response due to its non-species specificity [14, 15].

In this study, we investigated the mechanism through which CATH-2 modulates NLRP3 activation in LPS-primed mice primary peritoneal macrophages. Our results show that CATH-2 promoted IL-1β maturation and secretion via the NLRP3 pathway. Furthermore, CATH-2 induced ASC oligomerization, the formation of ASC specks and caspase-1 activation, leading to NLRP3 inflammasome activation. This process depended on K^+^ efflux. In addition, NEK7 and cathepsin B were also required for CATH-2-induced NLRP3 inflammasome activation. Our study reveals a novel modulatory role of CATH-2 and provides the basis for the development of novel therapeutic immunomodulatory strategies from the host perspective against antibiotic-resistant pathogens for cross species application.

## Materials and methods

### Animals

The wild-type (WT) C57BL/6 mice were purchased from Chongqing Academy of Chinese Material Medica (Chongqing, China). Nlrp3^−/−^, Asc^−/−^ and Casp1^−/−^ mice were a kind gift from Dr. Feng Shao, the NIBS (National Institute of Biological Sciences, Beijing, China). All gene knockout mice were on a C57BL/6 background and maintained in Specific Pathogen Free (SPF) conditions before being used at 8–10 weeks of age. All the animal experiments were approved by the Southwest University Ethics Committee, Chongqing, China (IACUC-2019-0627-05).

### Peptides

All the peptides were synthesized by China Peptides (Shanghai, China) using Fmoc-chemistry. All peptides were purified by reverse phase high-performance liquid chromatography to a purity > 95%.

### Preparation of macrophages and LPS stimulation in vitro

Mice were injected intraperitoneally with 2–3 mL of 4% thioglycolate medium (Eiken, Tokyo, Japan). After 3–4 days, the mice peritoneal exudate cells were collected by peritoneal lavage and suspended with RPMI 1640 supplemented with 10% FCS or Opti-MEM (Gibco, USA) as reported previously [21]. Then, the cells were seeded at 2 × 10^5^ cells/well for 48-well plates or 1 × 10^6^ cells/well for 12-well plates. These cells were maintained at a humidified 37 °C incubator with 5% CO_2_. After 2 h incubation, the nonadherent cells were removed and the adherent cells were used for assays described below. Subsequently, cells were stimulated with *E. coli* LPS (50 ng/mL) (Beyotime, China) for 3 h. After stimulation, peptides (5 μM) were added for an additional 21 h and ATP (1.5 mg/mL) (Beyotime, China) was added as the positive control. After 24 h, supernatants and cell lysates were collected for the assays described below. To inhibit K^+^ efflux and P2X7 receptor, KCl (5 mM and 50 mM) and P2X7 inhibitor (A-740003, 100 μM) (MedChemExpress, USA) were added after LPS stimulation. To inhibit CATH-2 endocytosis pathway, bafilomycin A1 (100 nM) and Cytochalasin B (10 μM) were added to incubate 1 h prior to CATH-2 treatment. Furthermore, to inhibit cathepsin activities, cathepsin B inhibitor (CA-074-Me, 20 μM) and cathepsin D inhibitor (pepstatin A, 20 μM) were added for 1 h incubation before LPS stimulation and CATH-2 treatment.

### Cell viability

Cells were prepared and treated as described above. After incubation, 10% WST-reagent was added according to the manufacturer’s protocols. Finally, absorbance was measured at 450 nm with a microplate reader (Bio-Rad, Japan) and was corrected for absorbance at 630 nm.

### Enzyme linked immunosorbent assay (ELISA)

Cells were prepared in 48-well plates and treated as described above. After incubation as described above, supernatants were collected and cytokine secretion was determined by ELISA according to the manufacturer’s instructions. The kits used in this study included IL-1β, TNF-α, IL-1α, IL-6 and IL-12 (Invitrogen, CA, USA).

### Western blot analysis

Cells were prepared in 12-well plates and treated as described above. After incubation as indicated, supernatants were collected and concentrated using 20% (w/v) trichloroacetate, and the cells were lysed with 1 × SDS loading buffer (Beyotime, China) and then cell lysates were collected. Subsequently, concentrated supernatants and cell lysates were subjected to 12% SDS-PAGE and then transferred onto a polyvinylidene difluoride (PVDF) membrane by electroblotting. Next, the membranes were blocked with 5% nonfat dry milk and then immunoblotted with the indicated antibodies (Abs) including anti-IL-1β (R&D, USA), anti-caspase-1 p20 (AdipoGen, USA), anti-pro-IL-1β, anti-pro-caspase-1, anti-NEK7 (Abcam, Cambridge, UK) and anti-β-actin (Beyotime, Beijing, China). Finally, the distinct protein bands were detected by ECL detection reagent (Biosharp, China).

### ASC oligomerization

Cells were prepared in 12-well plates and treated as described above. After incubation, cells were lysed with cold PBS containing 0.5% Triton X-100 and then cell lysates were centrifuged at 13 000 rpm for 15 min at 4 ℃ to obtain the cell pellets. The pellets were washed twice with cold PBS and suspended in 200 μL PBS. The resuspended pellets were cross-linked with 4 mM fresh disuccinimidyl suberate (DSS) at 37 ℃ for 30 min and then the pellets were centrifuged at 13 000 rpm for 15 min at 4 ℃. Finally, the cross-linked pellets were dissolved in 30 μL 1 × SDS-PAGE sample loading buffer and samples were boiled for 5 min before the Western blot analysis.

### Quantitative real time polymerase chain reaction (RT-PCR)

Cells were prepared in 12-well plates and treated as described above. After 6 h incubation, cells were lysed and total RNA were extracted by TRIzol Reagent (Life Technologies Carlsbad, CA, USA) according to the manufacturers’ instructions. Then, cDNA was synthesized using PrimeScript® RT reagent Kit (Perfect Real Time) (Takara, Japan). Finally, RT-PCR was performed using the CFX96 (Bio-Rad, USA). Primers were used as follows: IL-1β forward 5′-GAA ATG CCA CCT TTT GAC AGT G and reverse 5′-TGG ATG CTC TCA TCA GGA CAG, NLRP3 forward 5′-CTT TCT GGA CTC TGA CCG GG and reverse 5′-CTC CCA TTC TGG CTC TTC CC, β-actin forward 5′-TGG AAT CCT GTG GCA TCC ATG AAA C and reverse 5′-TAA AAC GCA GCT CAG TAA CAG TCC G. Relative gene expression levels were normalized against the expression levels of β-actin.

### SiRNA interference

Cells were prepared as described above and transfected using Lipofectamine 3000 (Thermo Scientific, USA) with 60 nM of Nek7 siRNA (Sangon Biotech, 5′-GAUAGACUGUGUUUAUAGATT-3′) or 60 nM of control siRNA (Sangon Biotech, 5′-UUCUCCGAACGUGUCACGUTT-3′) for 48 h. Subsequently, cells were primed with LPS and treated with CATH-2 as described above. After incubation, cell supernatants and lysates were collected for ELISA and Western blot analysis.

### Immunofluorescence staining

Cells were prepared in 48-well plates and treated with LPS and CATH-2 as described above. After 6 h incubation, cells were washed three times with PBS and fixed in 4% paraformaldehyde (Sango Biotech, Shanghai, China) for 20 min at room temperature (RT). After three wash steps, cells were permeabilized with 0.1% Triton X-100 in PBS for 10 min. Subsequently, cells were blocked with 5% Bovine Serum Albumin (BSA) in PBS for 30 min. Then, cells were stained with primary antibody containing anti-NLRP3 (Bioss, Beijing, China) and anti-ASC (Santa cruz, CA, USA) for 1 h at RT. After the wash steps, cells were incubated with Goat anti-mouse IgG (H&L) Alexa fluor 488 and Goat anti-rabbit IgG (H&L) Alexa fluor 594 (Abcam, UK) for 1 h. DAPI (Beyotime Biotechnology, Shanghai, China) was added for 5 min to visualize cell nuclei. Finally, cells were washed and maintained in antifading medium (Solarbio, Beijing, China). Cells were observed using the fluorescence microscopy (Olympus, Tokyo, Japan).

### Lysosomal leakage

Cells were prepared in 48-well plates as described above. Then, cells were loaded with Alexa 488 Dextran (20 μM), 10 kDa (Life Technologies) for 3 h prior to LPS and CATH-2 treatment for further 6 h. After incubation, the cells were observed using fluorescence microscopy (Olympus, Tokyo, Japan).

### Statistical analysis

Data are represented as mean ± SEM of three independent experiments for each group (*n* = 3). One-way ANOVA were used to analyze statistical significance among different groups. Statistical significance was shown as *p < 0.05, **p < 0.01, ***P < 0.001, ns = no significance.

## Results

### CATH-2 induces IL-1β and IL-1α secretion in LPS-primed macrophages

**Figure 1:**
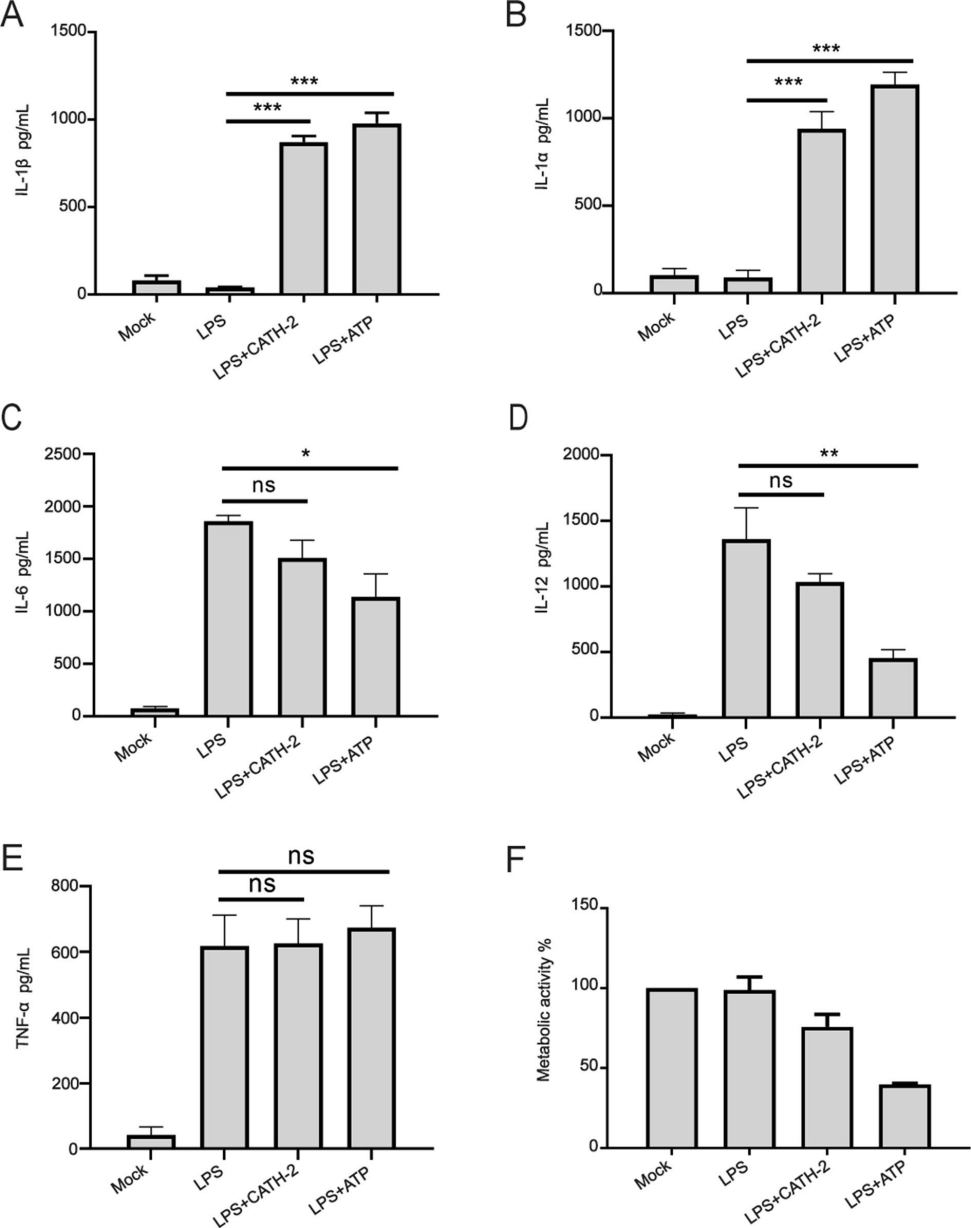
**CATH-2 induces IL-1β and IL-1α secretion in LPS-primed macrophages.** Cells were primed with LPS for 3 h and then CATH-2 (5 μM) and ATP (1.5 mg/mL) as positive control were added for an additional 21 h. After 24 h, supernatants were collected to determine cytokine secretion of IL-1β (**A**), IL-1α (**B**), IL-6 (**C**), IL-12 (**D**) and TNF-α (**E**) using ELISA; WST-1 was used to detect metabolic activity of cells (**F**). Data are represented as mean ± SEM of three independent experiments of triplicate samples per experiment. *P ≤ 0.05; ** *P* ≤ 0.01; *** *P* ≤ 0.005.

To investigate immunomodulatory properties of CATH-2 after LPS stimulation, different cytokines were measured in mice primary peritoneal macrophages. As shown in Figure 1, LPS induced high levels of IL-6, IL-12 and TNF-α secretion in macrophages but it failed to induce IL-1β and IL-1α secretion (Figures 1A, B, C). Under the treatment of CATH-2 and ATP, IL-1β and IL-1α were produced in LPS-primed macrophages while IL-6, IL-12 and TNF-α production was not affected by CATH-2. However, ATP significantly decreased LPS-induced production of IL-6 and IL-12 (Figures 1C, D). Moreover, CATH-2 had no effect on cell viability but ATP significantly reduced cell viability (Figure 1F), suggesting that ATP induces cell death. These results indicate that CATH-2 specifically acts on the IL-1β signaling pathway.

### CATH-2 induces NLRP3 activation via ASC oligomerization and caspase-1 activation in LPS-primed macrophages

**Figure 2:**
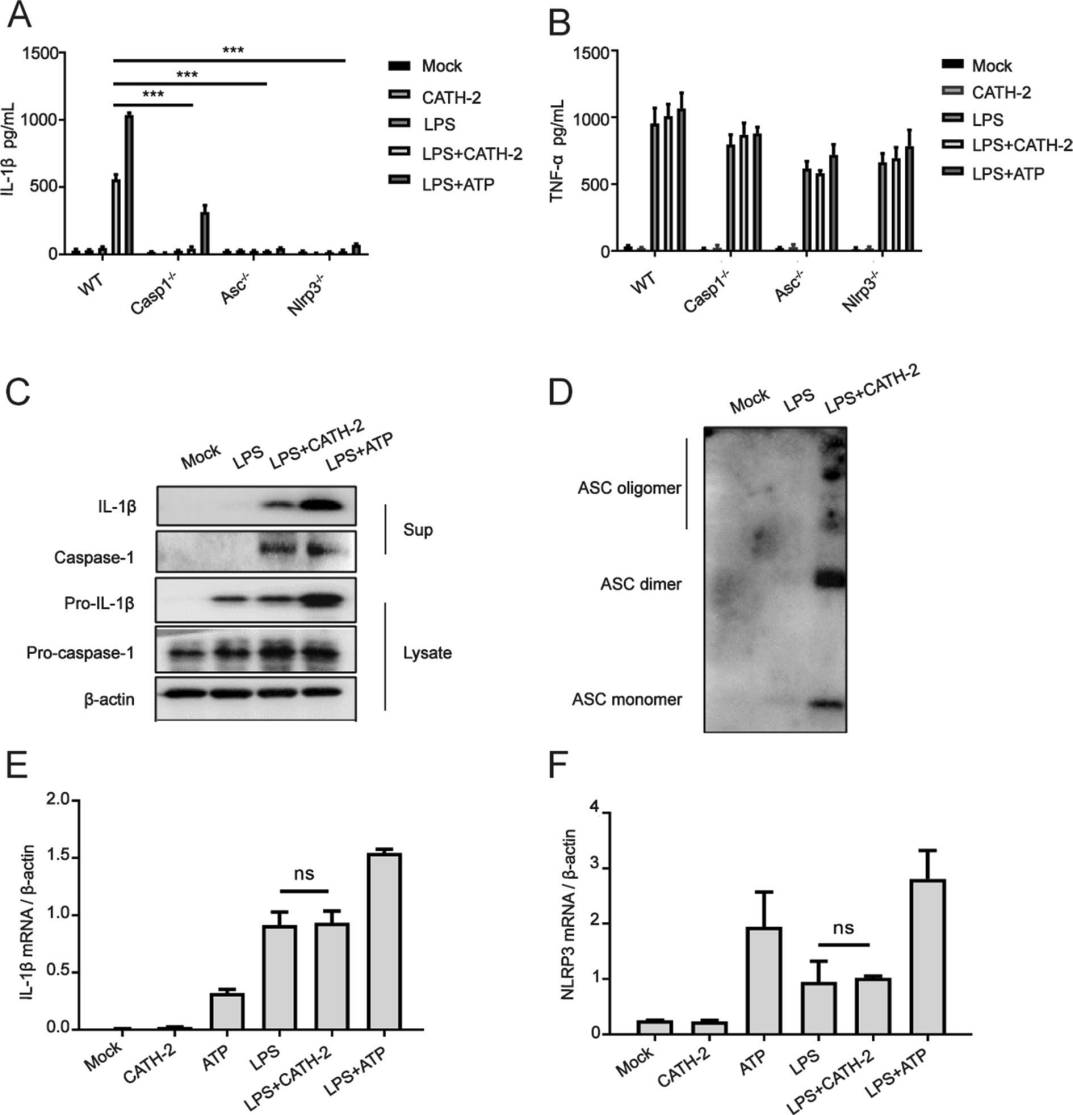
**CATH-2 induces NLRP3 activation via ASC oligomerization and caspase-1 activation in LPS-primed macrophages.** Cells from WT, Nlrp3^−/−^, Asc^−/−^, Casp1^−/−^ mice were primed with LPS for 3 h and then CATH-2 (5 μM) and ATP (1.5 mg/mL) as positive control were added for an additional 3 h or 21 h. After 24 h, cell supernatants and lysates were collected for different assays. ELISA was used to determine cytokine secretion of IL-1β (**A**) and TNF-α (**B**); A Representative image of the Western blot assay is presented to show protein expression including IL-1β, caspase-1, pro-IL-1β, pro-caspase-1 and β-action (**C**) as well as ASC protein expression (**D**); qPCR was used to measure mRNA expression of IL-1β (**E**) and NLRP3 (**F**). Data in **A**, **B**, **D**, **F** are represented as mean ± SEM of three independent experiments from triplicate samples per experiment. * *P* ≤ 0.05; ** *P* ≤ 0.01; *** *P* ≤ 0.005.

NLRP3 activation leads to IL-1β secretion via the assembly of adaptor protein ASC to recruit caspase-1 which cleaves pro-IL-1β. To further investigate whether CATH-2 induced IL-1β secretion via the NLRP3 pathway, macrophages from WT, Nlrp3^−/−^, Asc^−/−^, and Casp1^−/−^ mice were used in this study. The results show that CATH-2 and ATP-induced IL-1β secretion was completely abrogated in LPS-primed Nlrp3^−/−^, Asc^−/−^, and Casp1^−/−^ macrophages while TNF-α secretion was not affected (Figures 2A, B), indicating CATH-2-induced IL-1β secretion via an NLRP3-dependent mechanism. Subsequently, caspase-1 and ASC oligomerization were detected by Western blot. The results show that CATH-2 induced caspase-1 activation and ASC oligomerization in LPS-primed macrophages (Figures 2C, D). However, CATH-2 did not promote LPS-induced mRNA expression of IL-1β and NLRP3 (Figures 2E, F). These results demonstrate that CATH-2 acts as a second signal to activate NLRP3, resulting in IL-1β maturation and secretion.

### CATH-2 induces NLRP3 activation via a P2X7R-independent manner but dependent on K^+^ efflux in LPS-primed macrophages

It has been reported that ATP-mediated activation of the P2X7 receptor (P2X7R), a ligand-gated ion channel of the purinergic receptor family, promotes NLRP3 activation dependent on K^+^ efflux [23]. According to the findings that the role of CATH-2 as a 2^nd^ signal to activate NLRP3 is similar to the role of ATP, we identified the effect of P2X7R and K^+^ efflux on CATH-2-induced NLRP3 activation in LPS-primed macrophages.

**Figure 3:**
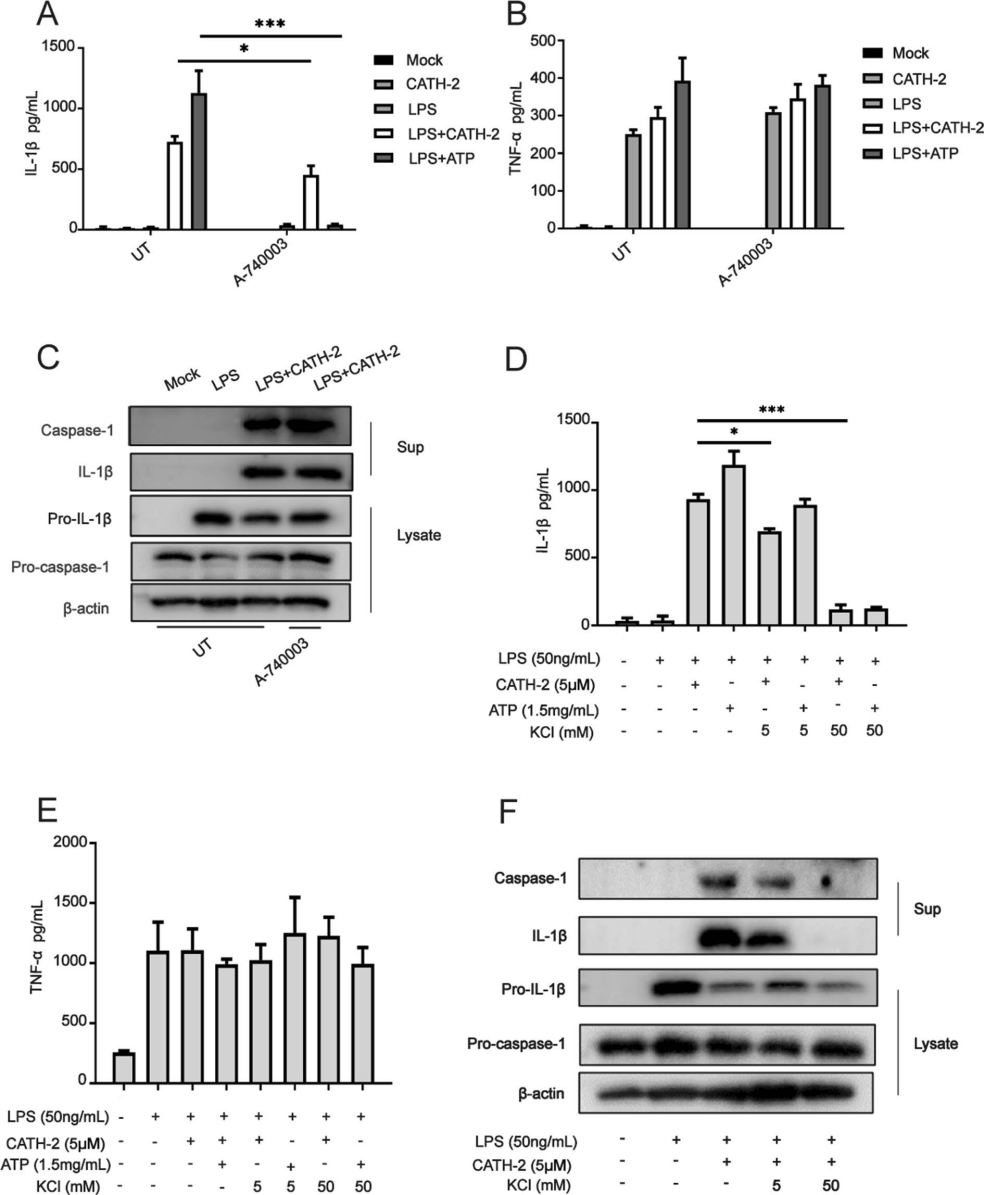
**CATH-2 induces NLRP3 activation via a P2X7R-independent manner but dependent on K**^+^** efflux in LPS-primed macrophages.** Cells were primed with LPS for 3 h and then CATH-2 (5 μM) and ATP (1.5 mg/mL) as positive control were added for an additional 21 h. To inhibit K + efflux and the P2X7 receptor, cells were untreated (UT) or treated with KCl (5 mM and 50 mM) and the P2X7R inhibitor (A-740003, 100 μM) after LPS stimulation. Finally, cell supernatants and lysates were collected for different assays. ELISA was used to determine cytokine secretion of IL-1β (**A**, **D**) and TNF-α (**B**, **E**). A representative image (Western blot) is presented to show protein expression including IL-1β, caspase-1, pro-IL-1β, pro-caspase-1 and β-action (**C**, **F**). Data in **A**, **B**, **D**, **E** are represented as mean ± SEM from three independent experiments with triplicate samples per experiment. * *P* ≤ 0.05; ** *P* ≤ 0.01; *** *P* ≤ 0.005.

To block these two signaling pathways, P2X7R inhibitor A-740003 and KCl were used in this study. The results show that the P2X7R inhibitor had no effect on CATH-2 induced IL-1β secretion in LPS-primed macrophages while the inhibitor completely abolished ATP-induced IL-1β secretion (Figure 3A). On the contrary, TNF-α secretion that is not related to inflammasome formation, was not affected by the P2X7R inhibitor (Figure 3B). Notably, the results of the Western blot analysis show that CATH-2-induced IL-1β and caspase-1 protein expression were not affected by the P2X7R inhibitor (Figure 3C), suggesting CATH-2-induced NLRP3 activation independent of the P2X7 receptor. In addition, after K^+^ efflux was blocked by KCl in LPS-primed macrophages, both CATH-2 and ATP-induced IL-1β secretion were significantly inhibited but TNF-α secretion was not affected (Figures 3D, E). Furthermore, CATH-2-induced caspase-1 activation was also attenuated by KCl (Figure 3F). These results indicate that CATH-2 promotes K^+^ efflux independently of P2X7R, which leads to NLRP3 activation.

### NEK7 is involved in CATH-2-induced NLRP3 activation in LPS-primed macrophages

**Figure 4:**
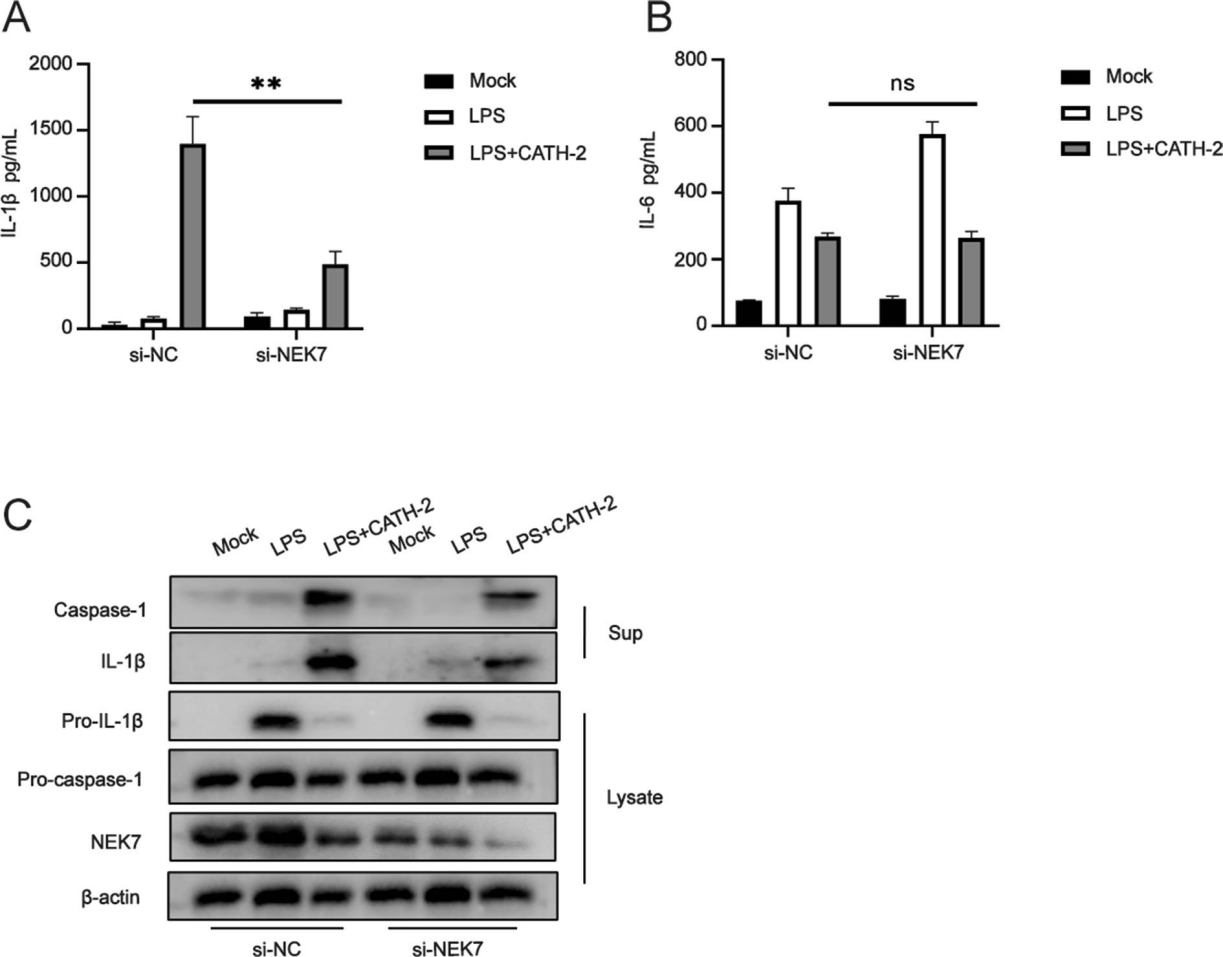
**NEK7 is involved in CATH-2-induced NLRP3 activation in LPS-primed macrophages.** Cells were transfected with NEK7 siRNA and control siRNA for 24 h. Subsequently, cells were primed with LPS for 3 h and then CATH-2 (5 μM) were added for an additional 21 h. Cell supernatants and lysates were collected for different assays. ELISA was used to determine cytokine secretion of IL-1β (**A**) and IL-6 (**B**). A representative image (Western blot) is presented to show protein expression including IL-1β, caspase-1, pro-IL-1β, pro-caspase-1 and β-action (**C**). Data in **A**, **B** are represented as mean ± SEM from three independent experiments with triplicate samples per experiment. * *P* ≤ 0.05; ** *P* ≤ 0.01; *** *P* ≤ 0.005.

NEK7 is a serine threonine kinase which is found to be critical for NLRP3 inflammasome activation and which specifically interacts with NLRP3 but not with the other inflammasomes [24]. To identify whether CATH-2-induced NLRP3 activation requires NEK7, its gene expression was silenced using siRNA knock-out. The results show that CATH-2 induced-IL-1β secretion was significantly decreased after NEK7 siRNA transfection in LPS-primed macrophages, which was consistent with the results in Western blot analysis (Figures 4A, C). IL-6 secretion was not affected by silencing of NEK7 confirming the specific role of NEK7 in NLRP7 inflammasome formation (Figure 4B). Moreover, CATH-2-induced caspase-1 protein expression was also significantly reduced in NEK7 knock out macrophages (Figure 4C). These results demonstrate that CATH-2 promotes NEK7-NLRP3 interaction, leading to NLRP3 inflammasome activation.

### Lysosomal leakage of cathepsin B mediates CATH-2-induced NLRP3 activation in LPS-primed macrophages

**Figure 5:**
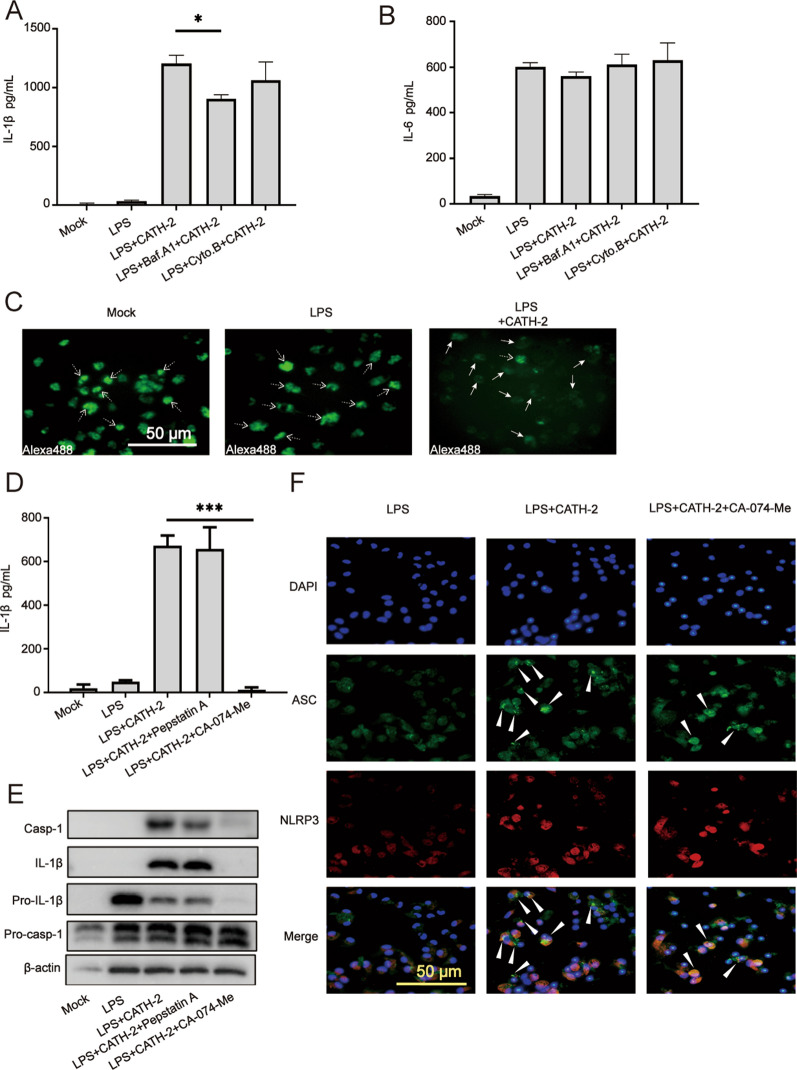
**Lysosomal leakage of cathepsin B mediates CATH-2-induced NLRP3 activation in LPS-primed macrophages.** Cells were primed with LPS for 3 h and then Baf.A1 (100 nM) and Cyto.B (10 μM) were added for 1 h incubation prior to CATH-2 treatment for a further 21 h. ELISA was used to determine cytokine secretion of IL-1β (**A**) and IL-6 (**B**). Confocal microscopy images of cells loaded with Alexa 488 Dextran (green) treatment with LPS and CATH-2, with dashed white arrows indicating fluorescent signal in lysosomes and solid white arrows indicating lysosomal leakage (**C**). Furthermore, cells were pretreated with cathepsin B inhibitor (CA-074-Me, 20 μM) and cathepsin D inhibitor (pepstatin A, 20 μM) for 1 h. Subsequently, cells were primed with LPS for 3 h and then CATH-2 (5 μM) was added for an additional 21 h. ELISA was used to determine cytokine secretion of IL-1β (D). A representative image of the Western blot assay is present to show protein expression including IL-1β, caspase-1, pro-IL-1β, pro-caspase-1 and β-action (**E**); Additionally, after 6 h incubation, immunofluorescence staining was performed using primary antibodies anti-NLRP3 and anti-ASC as well as secondary antibodies Goat anti-mouse IgG (H&L) Alexa fluor 488 and Goat anti-rabbit IgG (H&L) Alexa fluor 594. Representative images of ASC, ASC specks (white arrows), NLRP3 and cell nucleus (**F**). Data from figure A are represented as mean ± SEM of three independent experiments of triplicate samples per experiment. * *P* ≤ 0.05; ** *P* ≤ 0.01; *** *P* ≤ 0.005.

To further investigate the mechanism by which CATH-2 exerts its function, bafilomycin A1 (Baf.A1) and cytochalasin B (Cyto.B) were used to inhibit the CATH-2 endocytosis pathway. The results show that Baf.A1 significantly decreased CATH-2-induced IL-1β secretion while Cyto.B did not affect IL-1β secretion (Figure 5A). However, IL-6 secretion was not altered by Baf.A1 and Cyto.B (Figure 5B). These results reveal that CATH-2 endosomal acidification is required for NLRP3 activation.

It has been reported that lysosomal disruption is crucial for NLRP3 activation. To investigate whether CATH-2 induces lysosomal leakage, fluorescently labelled Dextran was loaded in the macrophages, which resulted in punctate accumulation of fluorescent signal in lysosomes (Figure 5C, dashed white arrows). Subsequent CATH-2 treatment significantly reduced punctate accumulation of the fluorescent signal in lysosomes, evidenced by the diffusion of the fluorescent signal (Figure 5C, solid white arrows), indicating the lysosomal leakage.

To further investigate the significance of leakage of lysosomal cathepsin B and D, cathepsin B inhibitor (CA-074-Me) and cathepsin D inhibitor (pepstatin A) were used to pretreat cells before LPS stimulation. The results show that the cathepsin B inhibitor abrogated CATH-2-induced IL-1β secretion while cathepsin D inhibitor did not affect CATH-2-induced IL-1β secretion (Figure 5D). Similarly, the cathepsin B inhibitor completely inhibited CATH-2-induced caspase-1 protein expression (Figure 5E). Early studies have revealed that polymerization of the adaptor ASC and the assembly of ASC specks recruit and activate caspase-1 [25]. Our immunofluorescent staining shows that CATH-2 induced formation of ASC specks (Figure 5F, white arrows) in LPS-primed macrophages. However, CATH-2-induced formation of ASC specks was also decreased by the cathepsin B inhibitor (Figure 5C, white arrows). These results indicate that CATH-2 promotes lysosomal leakage to release cathepsin B and then induces assembly of ASC specks, which results in NLRP3 inflammasome activation.

## Discussion

Cathelicidins exhibit potent antimicrobial activity against a broad spectrum of pathogens, such as bacteria, parasites, viruses and fungi [7, 26]. An additional function of cathelicidins is to modulate the host immune response, which contributes to host immune homeostasis [27]. The immunomodulatory activity of human cathelicidin LL-37 has been extensively studied. For example, LL-37 promotes cell differentiation, enhances killing and clearance of bacteria and regulates the production of inflammatory mediators in different cell types [27–31]. These responses are modulated by cell surface receptors including FPR2/ALX/P2X7R [27, 29]. However, the biological role of other cathelicidins such as the ones present in livestock, avian and fish is rarely studied. An exception is probably the chicken cathelicidin-2 for which the biological activities have also been extensively studied. Except for strong antimicrobial activity, CATH-2 to some extent shows partially similar immunomodulatory activities to LL-37 in human and mouse cells. Importantly, this function of CATH-2 has not been found to be species specific. CATH-2 has for example been found to play a role in LPS neutralization and modulation of TLR, but whether CATH-2 is involved in the modulation of intracellular signaling pathways remains unknown. Therefore, in this study, we explored the mechanism by which CATH-2 regulates NLRP3 inflammasome activation in mice peritoneal macrophages primed with LPS.

The NLRP3 inflammasome is the protein complex that recognizes different pathogens including bacteria and viruses, which plays an important role in the induction of an inflammatory response [17]. It has been reported that LL-37 internalizes into macrophages to induce the NLRP3 inflammasome [22]. Similarly, our study shows that CATH-2 activates NLRP3 inflammasome through the induction of ASC oligomerization and caspase-1 activation in LPS-primed macrophages. Furthermore, CATH-2 does not affect NLRP3 and IL-1β transcription. These results indicate that CATH-2 has similar immunomodulatory properties as LL-37 and both of them can act as a second signal to activate the NLRP3 inflammasome. Importantly, it has been reported that LL-37 activates NLRP3 inflammasome to generate protective epithelial cell inflammatory responses against *Pseudomonas aeruginosa* infection [32]. Due to the similar property with LL-37, it is reasonably speculated that CATH-2 could be used to enhance protective inflammatory responses for the clearance of immune tolerant pathogens.

The P2X7 receptor is highly expressed in macrophages and mediates the influx of Ca^2+^, Na^+^ and K^+^ ions, which controls the release of proinflammatory cytokines [17]. Our data show that ATP induces NLRP3 activation via the P2X7 receptor, which is consistent with the knowledge that ATP induces K^+^ efflux to activate the NLRP3 inflammasome [33]. Unlike LL-37’s internalization via the P2X7 receptor to activate the NLRP3 inflammasome in macrophages [23], our study shows that CATH-2 induces NLRP3 activation independently of the P2X7 receptor. However, some researchers have identified that the endocytosis of LL-37 via the P2X7 receptor depends on the cell type studied. In PBMC and epithelial cells, the P2X7 receptor was not actually involved in LL-37 internalization [32, 34]. In addition, Coorens et al. found that CATH-2/DNA complexes can be taken up by macrophages in a clathrin-dependent manner, providing clear evidence that CATH-2, at least in complex with DNA is endocytosed [13]. Combined with our results, it can be speculated that CATH-2 is internalized by macrophages and can then activate the NLRP3 pathway. However, the exact mechanism of CATH-2 endocytosis by macrophages needs to be further studied.

Efflux of K^+^ is thought to be a common trigger for NLRP3 inflammasome activation [33]. However, several reports have shown that NLRP3 inflammasome activation is independent of K^+^ efflux, possibly depending on the exact species that is studied as well [35]. In human monocytes, LPS-triggered NLRP3 inflammasome activation relies on NLRP3-ASC-caspase-1 signaling but is independent of K^+^ efflux [36]. In addition, imiquimod and CL097-mediated NLRP3 inflammasome activation directly targets mitochondria instead of induction of K^+^ efflux [36]. In contrast with these findings, our study demonstrates that the addition of extracellular high concentrations of KCl blocks efflux of K^+^ and thereby significantly inhibits CATH-2-induced NLRP3 inflammasome activation, indicating that CATH-2 might induce efflux of K^+^. Recent research has shown that K^+^ efflux is mediated by the K^+^ channel 2 (TWIK2) and the chloride intracellular channel (CLIC) protein acts downstream of K^+^ efflux to promote Cl^−^ efflux, leading to NLRP3 inflammasome activation [37, 38]. However, whether CATH-2 interacts with these proteins that control ion channels needs to be further studied.

Recently, NEK7 was proposed as an essential component of the NLRP3 inflammasome where it regulates assembly and activation of the inflammasome [39–41]. It has been identified that NEK7 protein binding to NLRP3 mediates the activation of the NLRP3 inflammasome. Some reports have shown that NEK7-NLRP3 interaction, as revealed by Co-IP and GST pull-down assays, can be induced by different stimuli, such as LPS + ATP, bacterial and pathogen toxins [24, 42, 43]. Although our data does not directly show NEK7-NLRP3 interaction, NEK7 knockdown significantly reduced CATH-2-induced caspase-1 activation and IL-1β secretion, indicating that CATH-2 might promote NEK7-NLRP3 interaction. However, it has been recently reported that K^+^ efflux regulates the NEK7-NLRP3 interaction [24]. Therefore, CATH-2-induced NLRP3 inflammasome activation might also be mediated by upstream signaling events of NLRP3 inflammasome.

Lysosomal disruption, as the upstream signal of NLRP3 inflammasome can also induce its activation [44–46]. Phagocytosis of particulates causes lysosomal acidification and rupture. In addition, studies that used cathepsin inhibitors indicate that cathepsins resident in the lysosomes are important to activate the NLRP3 inflammasome in response to external stimuli. Considering the endosomal uptake of CATH-2 it is possible that (at least part) of the role of CATH-2 in inflammasome activation lies in its possible role in disruption of lysosomes, thereby releasing cathepsins [13]. This is in line with the observation from our study that CATH-2-induced IL-1β secretion is required for CATH-2 endosome acidification and CATH-2 induced lysosomal leakage. In addition, CATH-2-induced IL-1β secretion is completely blocked by cathepsin B inhibitors, although this inhibition might also result from suppressed pro-IL-1β synthesis. Actually, it has been reported that cathepsin B-deficient macrophages have similar IL-1β secretion as normal macrophages [45], indicating that IL-1β secretion is independent of cathepsin B. However, CATH-2-induced caspase-1 activation and formation of ASC specks inhibited by cathepsin B inhibitors suggest that CATH-2-induced NLRP3 inflammasome activation is mediated by lysosomal leakage of cathepsin B. This finding is similar to the effect of LL-37 [32]. It will be interesting to discover the basis of these seemingly contradicting results on the involvement of Cathepsin B, but it is likely that differences in species or cell types studied, or even experimental setup can have an effect on the observations. Our results at least, reveal that cathepsin B is not only involved in the priming step of pro-IL-1β synthesis, but also mediates NLRP3 inflammasome activation.

In conclusion, CATH-2 acts as a second signal to activate the NLRP3 inflammasome in LPS-primed macrophages, leading to ASC oligomerization and the formation of ASC specks as well as caspase-1 activation, finally resulting in IL-1β maturation and secretion. This activation process is mediated by K^+^ efflux. Furthermore, NEK7 and cathepsin B are also involved in CATH-2-induced NLRP3 inflammasome activation. Our study provides new insight on the immunomodulatory activity of CATH-2 and contributes to the development of CATH-2 as an immunomodulatory adjuvant against microbial infection not only in chickens, but also in other mammalian species.

## Data Availability

All data sets generated for this study are included in the article.
